# Comparative effects of temporary anchorage devices combined with various auxiliary attachments on maxillary molar mesialization with clear aligners: a finite element analysis

**DOI:** 10.1186/s12903-026-08187-9

**Published:** 2026-04-01

**Authors:** Feiyu Wang, Yuxin Fan, Mingyuan Liu, Qingnan Mou, Ying Yang, Jianbo Gao, Yuxia Hou, Panjun Pu

**Affiliations:** 1 Key Laboratory of Shaanxi Province for Craniofacial Precision Medicine Research, College of Stomatology, Xi’an Jiaotong University, Xi’an, Shaanxi PR China; 2 Department of Orthodontics, College of Stomatology, Xi’an Jiaotong University, Xi’an, Shaanxi PR China; 3 Shaanxi Key Laboratory of Intelligent Robots, Xi’an Jiaotong University, Xi’an, Shaanxi PR China; 4 Department of Orthopaedics, Xijing Hospital, Fourth Military Medical University, Xi’an, PR China

**Keywords:** Clear aligners, Finite element analysis, Micro-implants, Molar mesialization

## Abstract

**Background:**

This study aimed to investigate the biomechanics of maxillary dentition, specifically displacement patterns, midline deviation, and stress distribution of the first molar and anchorage teeth during first molar mesialization using clear aligners (CA) assisted by temporary anchorage devices (TADs) combined with different auxiliary attachments, including buccal buttons, aligner-based angel buttons and power arms.

**Methods:**

Finite element models of the CA, micro-implants, maxillary teeth, periodontal ligament (PDL), and alveolar bone were constructed. Four finite element models were simulated: model A (CA alone, control); model B (CA with micro-implants connected to aligner-based angel buttons); model C (CA with micro-implants connected to buccal buttons on the first molar); and model D (CA with micro-implants connected to power arms on the buccal surface of the first molar). The simulations evaluated three-dimensional tooth displacement, midline deviation, tipping angles, and PDL hydrostatic pressure of the maxillary dentition.

**Results:**

Mesialization of the first molar using CA alone resulted in mesiolingual tipping and intrusion, while anchorage teeth experienced distobuccal or distolingual tipping and extrusion. Midline deviation tended toward the edentulous side due to reciprocal forces. Auxiliary force systems generally improved the efficiency of molar mesial movement from 44.12% to 52.92% and reduced anchorage tooth displacement compared to the control. Aligner-based angel buttons showed the most effective anchorage stability and minimal midline deviation (0.0137 mm). Buccal buttons produced the largest mesial movement of the first molar, with pronounced tipping (0.714°) and uneven PDL stress. Power arms showed near-bodily movement of the first molar and relatively uniform PDL stress, although anchorage control was less pronounced than that of aligner-based angel buttons.

**Conclusion:**

The combined use of micro-implants and auxiliary devices demonstrated distinct effects on the first molar mesialization and anchorage teeth stability. Notably, aligner-based angel buttons provided the most effective anchorage control and the least midline deviation, while power arms resulted in more bodily movement of the first molar.

**Supplementary Information:**

The online version contains supplementary material available at 10.1186/s12903-026-08187-9.

## Background

Clear aligner therapy (CAT) has gained significant popularity among both patients and orthodontists, primarily due to its enhanced comfort, superior aesthetics, and the ability to deliver a personalized and removable treatment approach [1, 2]. As the demand for CAT continues to rise, the importance of accurately predicting orthodontic forces becomes increasingly critical [3]. Precise force prediction not only facilitates controlled and effective tooth movement, but also enhances treatment efficiency, improves patient satisfaction, and contributes to optimal clinical outcomes.

The absence of the second premolar is commonly observed in clinical practice, often resulting from factors such as severe dental caries or advanced periodontal disease. The alternative treatments for the missing second premolars include prosthodontic treatment [4] and space closure through orthodontic treatment which involves the mesial movement of the molars [5]. Moreover, premolars are also the preferred extraction sites when necessary in orthodontics. For patients with mild dental crowding and a well-balanced facial profile, extraction of the second premolars is often considered a favorable option [6]. In such cases, space closure is typically achieved through the mesial movement of molars. However, it is well recognized that clear aligner (CA) has limitations in effectively controlling mesial tooth movement, especially when large mesial displacement of molars is required. Mesial movement is typically achieved by reducing the length of CA, which applies pressure to the posterior teeth. Without proper anchorage or auxiliary mechanics, molars tend to exhibit uncontrolled movement, such as mesial tipping and rotation [7]. In addition, anchorage loss is unavoidable due to the reciprocal forces generated on the anchorage teeth during molar mesialization, posing a challenge for correction in the later treatment stages [8]. Therefore, maintaining proper control of anchorage and molar movement during CAT becomes a critical factor for achieving successful orthodontic outcomes.

Many studies have shown that temporary anchorage devices (TADs) and auxiliary attachments can improve the efficiency of CAT. TADs are often used to assist with molar distalization, anterior retraction, and molar mesialization, in order to reduce anchorage loss [9–11]. Clinically, this is commonly achieved using auxiliaries such as precision cuts, buttons, or power arms, often in conjunction with elastics [12]. Different types of auxiliaries may affect the treatment efficiency of CA. Liu et al. [13] showed that connecting micro-implants to precision cuts, compared to buttons, can enhance the efficiency of molar distalization while reducing undesirable side effects such as proclination of the anterior teeth. However, when using CA for molar mesialization, the biomechanics differ from molar distalization [14]. Moreover, existing studies primarily focus on molar distalization, and evidence regarding the predictability and effectiveness of mesial movement of maxillary molars remains limited and inconclusive. Further clinical research and biomechanical evaluations are essential to improve our understanding of aligner-driven mesialization and to develop more effective strategies for achieving stable and controlled tooth movement.

Finite element analysis (FEA) is a robust and noninvasive computational technique used to approximate the mechanical behavior of complex geometries subjected to external forces with high accuracy. By discretizing complex structures into finite elements and solving mathematical models, FEA enables the prediction of stress, deformation, and failure points with high precision [15]. Using 3D modeling and simulation, it predicts tooth movement and CA performance, enabling personalized treatment plans for optimal results [16]. Few FEA studies have systematically investigated the biomechanics of CA-mediated molar mesial movement. The work by Lyu [17] established a foundational model simulating mandibular molar displacement using CA with auxiliary attachments. However, their study was limited to examining only molar displacement trends in auxiliary device-based orthodontics, excluding micro-implants and, more importantly, lacking a systematic biomechanical comparison of TADs combined with multiple auxiliary attachments. Therefore, establishing FEA models is essential to accurately simulate the biomechanics involved in micro-implants assisted molar mesialization. These models serve as a critical tool for predicting tooth movement and evaluating stress distribution under complex force systems. In this study, we focused on CAT incorporating micro-implants and auxiliary appliances to support maxillary molar mesialization.

To our knowledge, this is the first study to systematically analyze and quantify the orthodontic tooth movements in molar mesialization with TADs and auxiliary attachments through FEA. The objective is to evaluate the displacement patterns and stress distribution in the maxillary dentition during first molar mesialization. Specifically, we hypothesize that micro-implants can enhance the efficiency of molar mesialization, and that different auxiliary attachments will result in distinct tooth movement characteristics and stress responses, with power arms expected to produce more bodily movement and less tipping compared to other devices, while angel buttons are the most effective for maintaining midline alignment. This study aims to provide insights for treatment planning, including the selection of traction methods and strategies for tooth movement.

## Materials and methods

### Three-dimensional (3D) model reconstruction

Imaging data from cone beam computed tomography (CBCT) were collected from a 25-year-old female with normal occlusion, Class I skeletal relationship, and average vertical facial pattern, exhibiting no craniofacial deformities or prior orthodontic treatment. The participant provided written informed consent before taking part in the study. This study was approved by the Ethics Committee of Hospital of Stomatology, Xi’an Jiaotong University (11 [2022] NO.051). The CBCT scan had a voxel size of 0.3 × 0.3 × 0.3 mm³. To simulate a clinical extraction scenario, the right maxillary second premolar was digitally removed to create a post-extraction model. The DICOM images were input into Mimics 21.0 (Materialise NV, Belgium) to create the initial geometry, where threshold-based segmentation (256–3321 Hounsfield units) was employed to accurately isolate the maxillary bone and individual tooth components.

**Fig. 1:**
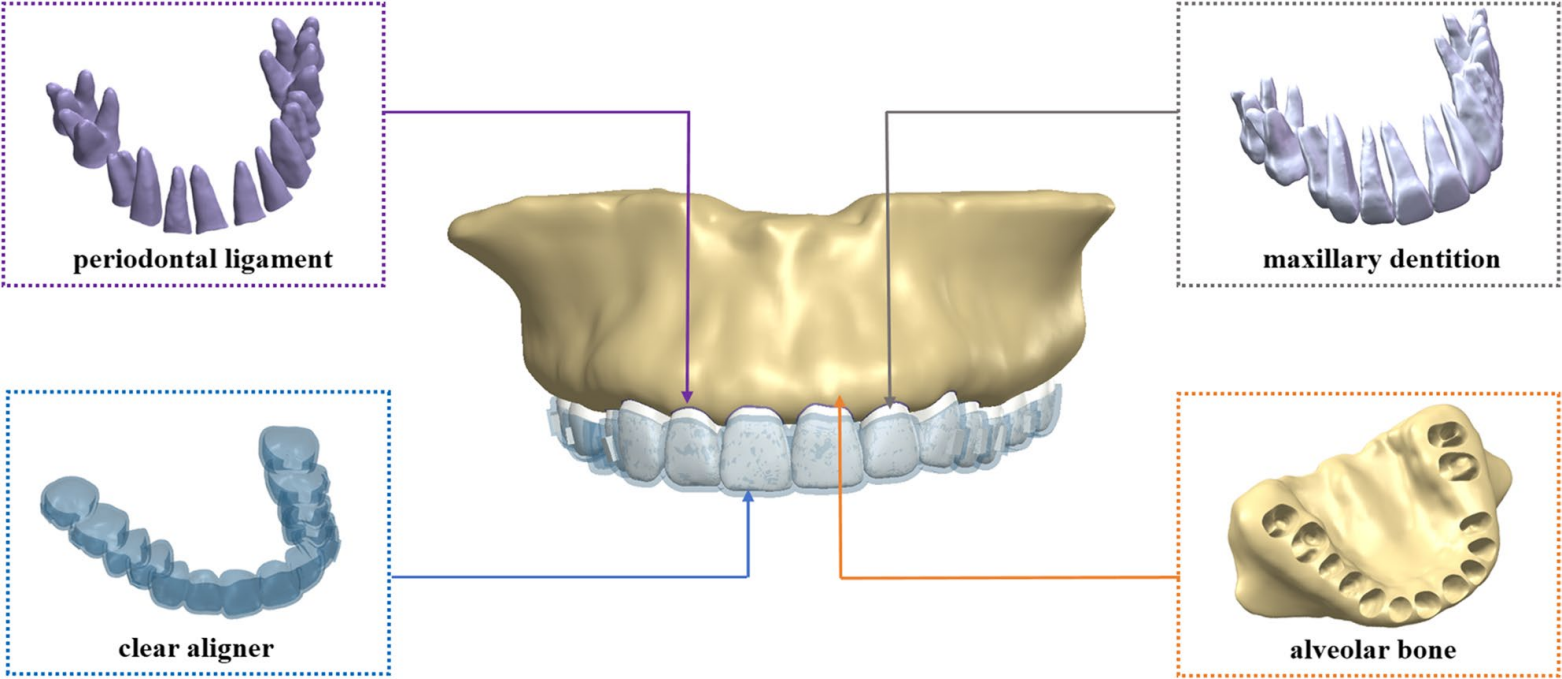
Structural elements of the model

Following segmentation, the preliminary 3D reconstructions were refined and smoothed using Geomagic Studio 2021 (3D Systems, North Carolina, USA) to eliminate noise and surface irregularities. The anatomical components, including the alveolar bone, periodontal ligament (PDL), and dentition, were then designed and assembled in SolidWorks 2024 (Dassault Systèmes, France) (Fig. 1). The PDL was modeled as a uniform layer 0.25 mm thick by offsetting the external surfaces of the tooth roots [18], reflecting its average physiological thickness and biomechanical properties.

To simulate clinical practice, attachments were incorporated into the model. The configurations of attachments from the canine to the second molar can enhance the efficiency of CA [19]. Vertical rectangular attachments (2 mm × 3 mm × 1 mm) were placed on the buccal surfaces of premolars and canines to enhance anchorage; horizontal rectangular attachments (3 mm × 2 mm × 1 mm) were applied to the buccal surfaces of the maxillary molars to enhance retention and reduce tipping movement during molar mesialization [20, 21]. The buttons (3 mm in diameter at the base and 1 mm in height) were placed on the first molar and CA. Power arms (3 mm in diameter at the base and 8 mm in height) were placed on the first molar. A micro-implant (diameter: 1.5 mm, height: 8 mm) representing skeletal anchorage was integrated into the model to assist in molar mesialization, positioned between the maxillary canine and the first premolar at a 60° angle to the occlusal plane and 6 mm apical to the alveolar crest. This placement was based on previous studies and modified to align with the current study [13, 22].

**Fig. 2:**
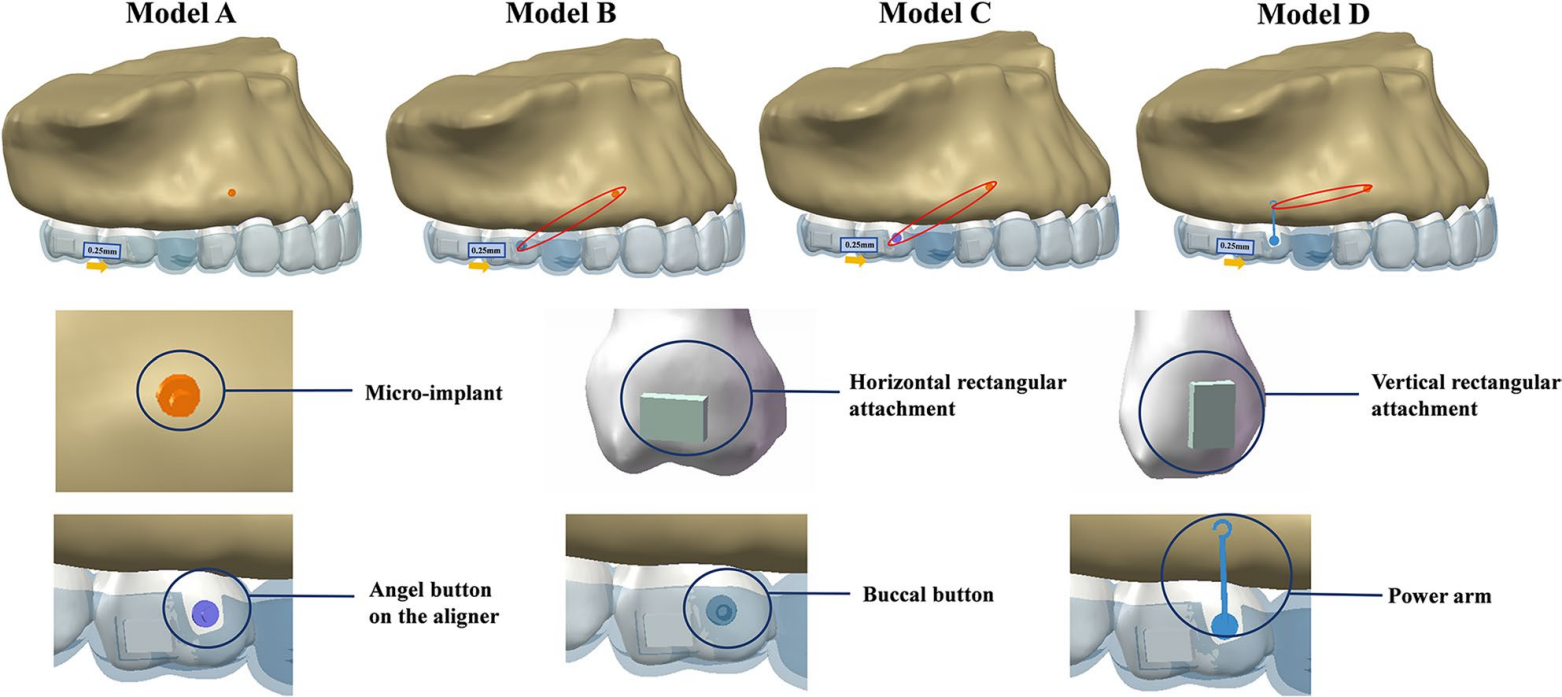
Submodels and groups. Models (A, B, C and D) were used to simulate the mesialization of the first molar by 0.25 mm distance. Model A was the control group. Model B represented aligner-based angel buttons combined with micro-implants. Model C used buccal buttons in combination with micro-implants. Model D represented power arms combined with micro-implants

To evaluate the biomechanical impact of diverse auxiliary designs during maxillary molar mesialization with CA, four distinct FE models were developed (Fig. 2):

#### Model A (Control group)

Simulated molar mesialization using CA alone, without any anchorage reinforcement or auxiliary devices. This group served as the baseline for comparison.

#### Model B (Aligner-based angel button group)

An angel button was incorporated into CA on the buccal surface of the first molar to enable the application of elastic forces by attaching external elastics to a micro-implant.

#### Model C (Buccal button group)

Included a standardized buccal button bonded to the buccal surface of the first molar. The button-tooth interface was modeled as a rigid bond to simulate clinical composite resin bonding.

#### Model D (Power arm group)

Included a standardized power arm structure bonded to the buccal surface of the first molar. The connection between the power arm and the tooth surface was simulated as a rigid bond.

Each model was subjected to identical boundary conditions and loading protocols to enable accurate comparison of force transmission, stress distribution, and predicted tooth displacement patterns under varying auxiliary mechanics.

### Material and mesh settings

The refined anatomical model and CA were loaded into ANSYS Workbench 2024 (Pennsylvania, USA) to construct a detailed FE model for biomechanical analysis. The model components, including the dentition, attachments, orthodontic buttons, PDL, and micro-implants were all defined as homogeneous, isotropic, and linearly elastic materials, consistent with parameters reported in previous literature [12, 23–25] (see Table 1 for specific material properties).

**Table 1:** Material properties

Material	Young’s modulus (MPa)	Poisson’s ratio
Teeth [23–25]	1.96 × 10^4^	0.3
Alveolar bone [23–25]	1.37 × 10^3^	0.3
PDL [25]	0.67	0.45
Attachment [23–25]	1.25 × 10^4^	0.36
Clear Aligner [24]	5.28 × 10^2^	0.36
Buttons [12]	1.14 × 10^5^	0.35
Micro-implants [12]	1.14 × 10^5^	0.35

All geometric models were discretized using 10-node tetrahedral (solid187) elements, which offered higher accuracy in capturing stress-strain behavior in complex anatomical structures. Table 2 presents the nodes and elements for each model. To enhance computational efficiency while maintaining numerical precision, region-specific mesh densities were applied based on the structural complexity and mechanical sensitivity of each component. The element sizes were defined as follows: 0.15 mm for the PDL, 0.20 mm for the dentition, orthodontic attachments, and appliances, and 0.25 mm for the maxillary bone [26]. These mesh settings were optimized through a convergence test to balance computational cost and solution accuracy.

**Table 2:** Nodes and elements

	Node no.	Element no.
Model A	819,903	1,624,312
Model B	844,085	1,670,167
Model C	852,797	1,683,491
Model D	851,934	1,682,678

### Boundary and contact conditions

A fixed support was applied to the superior portion of the maxilla to stabilize the finite element model during orthodontic force simulation, with all translational and rotational degrees of freedom fully constrained. This constraint effectively prevented undesired movement or deformation of the maxillary base during the application of orthodontic loading, thereby preserving the structural integrity of the model throughout the simulation process. Additionally, tie contact relationships were established between biologically bonded or continuous anatomical structures to simulate perfect bonding with no relative motion. These included the interfaces between the tooth and the PDL, the PDL and the alveolar bone, the alveolar bone and the micro-implant, as well as between the orthodontic attachment, the buccal button, and the tooth surface. These tie constraints allowed for accurate transmission of force and stress across the different tissue types. For components that exhibited contact under force with potential sliding, surface-to-surface contact elements were defined. A frictional coefficient of 0.2 was applied to the interface between the CA and the attachments, as well as between the CA and the tooth surfaces [23]. The frictional contact settings allowed for realistic modeling of tooth displacement and force dissipation during tooth movement.

### Loading methods and coordinate system

A static loading condition was adopted for the FE simulation to replicate the force system applied during each step of molar mesialization with CA. The mesial movement of the maxillary first molar was simulated by applying a single, prescribed mesial displacement of 0.25 mm with CA, simulating one step of clinical CA activation. In clinical orthodontics, forces in the range of 100-200 g are commonly used to assist tooth movement. A 100 g force generates lower stress on the PDL, resulting in slower tooth movement, which benefits periodontal health [27]. A 150 g force is a moderate force commonly used in anterior retraction [28]. A 200 g force exerts higher stress on the PDL, accelerating tooth movement, often used for molar distalization, but it may increase the risk of root resorption [29, 30]. The selection of orthodontic force magnitudes depends on factors such as the type of tooth movement, PDL area, and PDL response. A series of forces of 100 g, 150 g, and 200 g was applied by a spring attached to the micro-implants and the buttons or power arms to imitate the force.

**Fig. 3:**
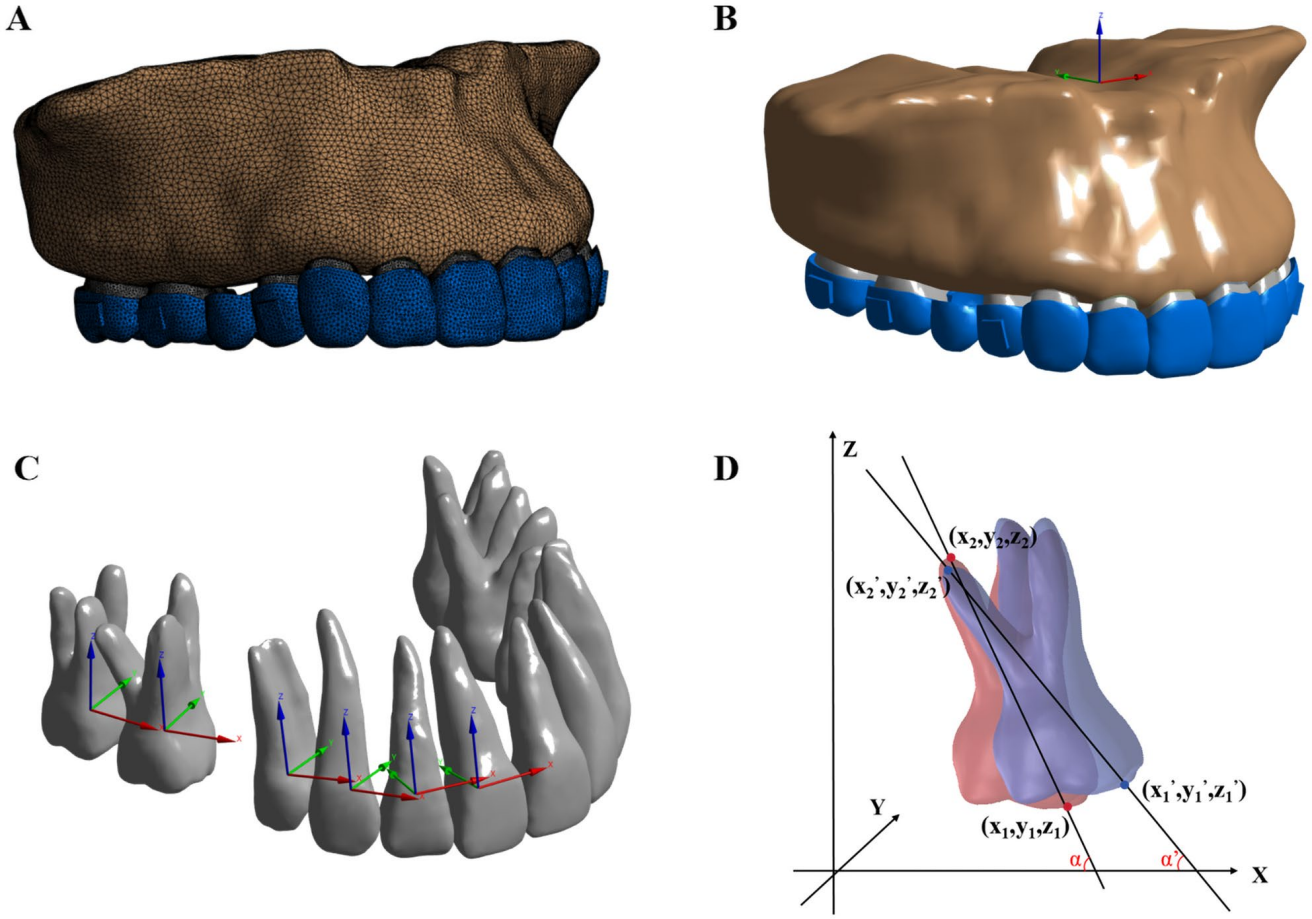
Mesh setup, coordinate systems, and tipping angle measurement. **A** Mesh setup; **B** Global coordinate system; **C** Local coordinate system; **D** Tipping angle measurement

Two coordinate systems were established for reference [21]. The global coordinate system was applied to the complete dental arch (Fig. 3B). The x-axis defined the coronal direction (left/right), the y-axis defined the sagittal direction (posterior/anterior), and the z-axis defined the vertical direction (superior/inferior). The local coordinate system was constructed for each tooth to quantify its three-dimensional displacement. In this system, the z-axis was defined as the long axis of the tooth representing the occlusogingival (or coronoapical) direction, the x-axis represented the mesiodistal direction, the y-axis corresponded to the buccolingual (or labiolingual) direction (Fig. 3C). Displacement vectors were recorded with the mesial, palatal, and gingival directions defined as positive (+) values, allowing for the precise evaluation of individual tooth movement patterns in response to orthodontic force application.

### Measurements

To analyze the first molar displacement trends, we measured the average displacement values from four cusp landmarks (mesiobuccal, distobuccal, and two palatal cusps) and three root apex points (mesiobuccal, distobuccal, and palatal roots) of the first molar, representing crown and root movement respectively. The x-axis and z-axis coordinates of the first molar were used to assess the sagittal tipping tendency. The rotation angles θ of the first molar in the standard anatomical plane (XZ) were defined in Eq. (2), the parameter α denoted the rotational change of the tooth, expressed in radians, and can be calculated using the given equation Eq. (1), as illustrated in Fig. 3D.

1 $$\triangle\alpha=\alpha'-\alpha=\arctan(\frac{Z_{2}-Z_{1}}{X_{1}-X_{2}})-\arctan(\frac{Z'_{2}-Z'_{1}}{X'_{1}-X'_{2}})$$

2 $$\theta=\frac{180^\circ}{\pi}\triangle\alpha$$

Midline deviation was determined using the global coordinate system by selecting the midpoint of the incisal edges between the maxillary central incisors (teeth 11 and 21). The mesial movement efficiency was calculated as the percentage of crown displacement in the x-axis direction to the designed 0.25 mm displacement, as shown in Eq. (3). The crown displacement was defined as the average displacement of the cusp tip. Other undesired movements, including extrusion or intrusion of teeth, crown tipping or rotation, lingual or buccal displacement, and midline deviation, occupied the remaining percentage.

3 $$P=\frac{X-axis}{0.25}$$

## Results

### The whole dentition displacement

**Fig. 4:**
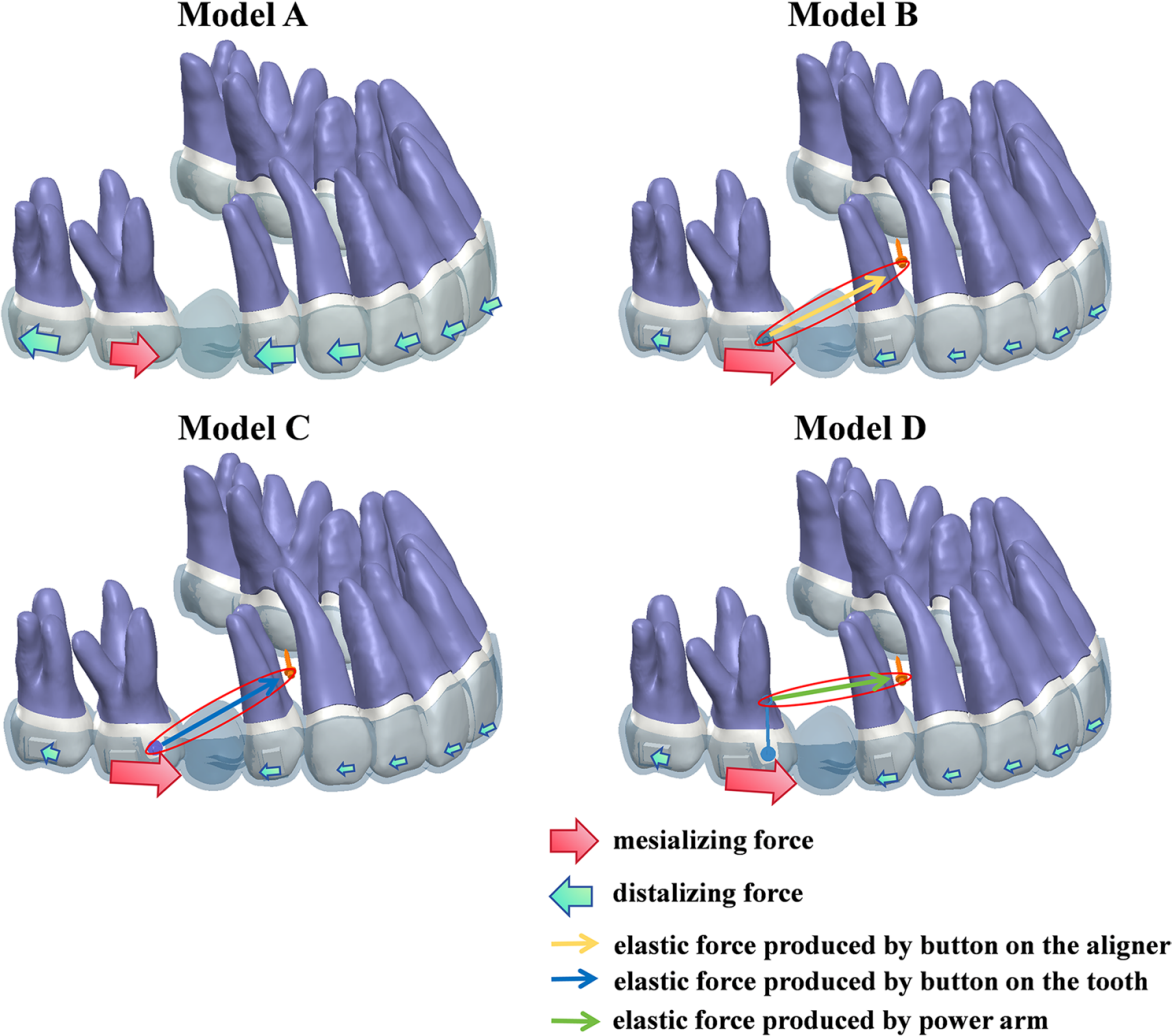
The force loading system. Mesial movement of the first molar generated reciprocal forces on adjacent teeth, which manifested as distal displacement of the second molar and distalization of the premolars and anterior teeth toward the edentulous area

**Fig. 5:**
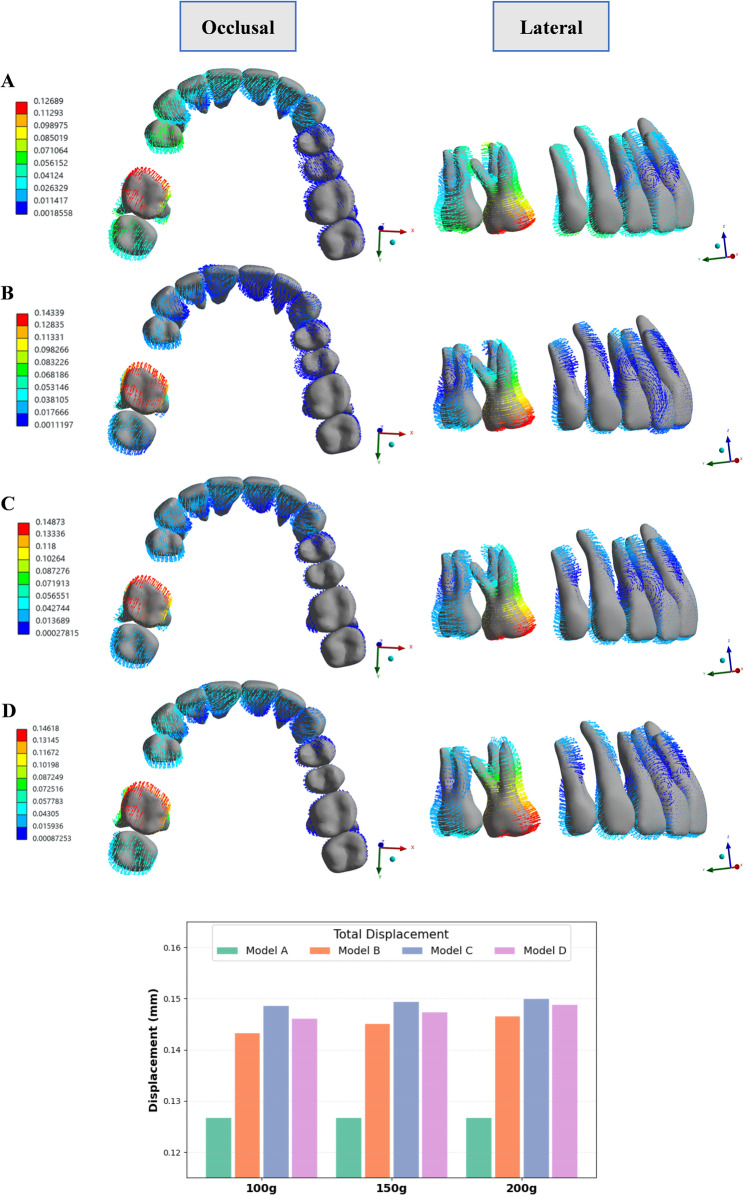
Total displacement of the maxillary dentition in four models. Model (**A**) (control, without anchorage reinforcement), model (**B**) (micro-implants connected to the tooth via an angel button), model (**C**) (micro-implants connected to a button which was bonded to the first molar), model (**D**) (micro-implants connected to a power arm which was bonded to the first molar). Displacement directions across the entire maxillary arch are illustrated using vector diagrams. Bar charts present the magnitude of total displacement for each model under different levels of traction force (mm). In this system, the x-axis corresponds to the coronal plane (positive to the left, negative to the right), the y-axis aligns with the sagittal plane (positive toward the posterior, negative toward the anterior), and the z-axis aligns with the vertical plane (positive in the superior direction, negative in the inferior direction). The distribution of displacement vectors demonstrated the mesial movement of the first molar and reciprocal distal movement of anchorage teeth, with increasing traction force producing larger total displacement magnitudes across all models

Figure 4 illustrated the force loading system exerted by CA. Model A used CA alone. Model B applied elastic force to the angel button. Model C delivered the elastic force directly to the first molar via a buccal button, while model D exerted the force on a power arm bonded to the buccal surface of the first molar. As the first molar shifted mesially, an opposing force was exerted on the anchorage teeth, causing them to move in the reverse direction. The use of auxiliary devices and micro-implants helped reduce anchorage loss. Figure 5 and Supplementary file 1 depicted the resultant displacement trends across the maxillary dentition. Across all experimental models, model C, featuring a buccal button on the first molar with 200 g of traction force, showed the maximal displacement of 0.1501 mm. In contrast, model A without micro-implants demonstrated minimal displacement of only 0.1269 mm.

**Fig. 6:**
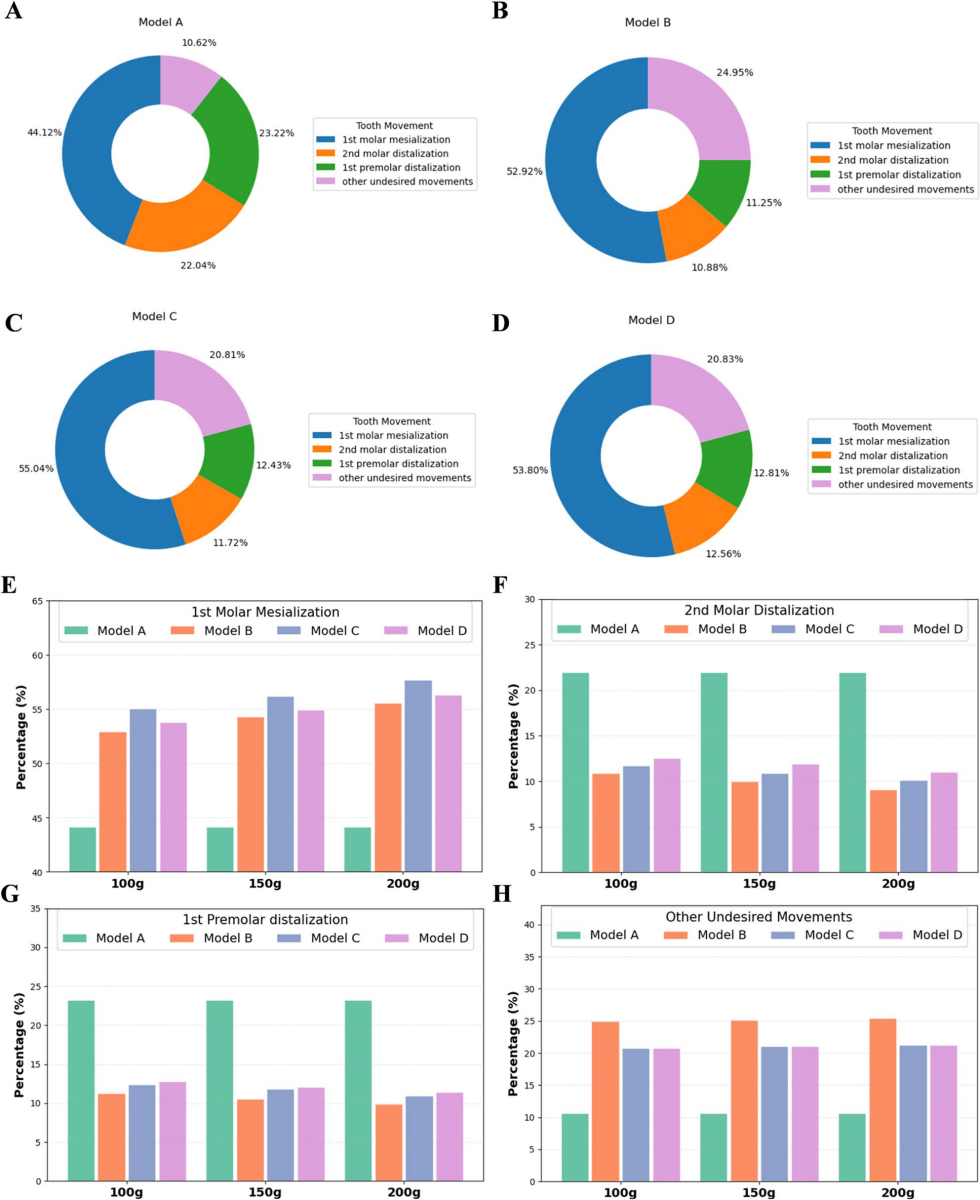
The ratio of effective teeth displacement contributing to a 0.25 mm mesialization of the first molar. The contribution of the first molar mesialization, anchorage teeth distalization, and other undesired movements (e.g., tipping and rotation, buccolingual displacement, extrusion or intrusion of anchorage teeth, and midline deviation) in models (**A**, **B**, **C** and **D**). **E **The contribution of the first molar mesialization among the four models under varying traction forces. **F** The contribution of the second molar distalization in the four models under varying traction forces. **G** The contribution of the first premolar distalization in the four models under varying traction forces. **H** The proportion of other undesired tooth movement among the four models under varying traction forces. The results indicated that micro-implant assisted auxiliaries (models B–D) achieved a higher proportion of effective molar mesialization compared with the control, whereas increasing traction force enhanced total movement efficiency but also increased the share of undesired movements

Figure 6 demonstrated the efficiency of tooth movement contributing to first molar mesialization at a 0.25 mm distance. In model A, the first molar achieved 44.12% of the intended mesialization, while the second molar and first premolar moved distally by 22.04% and 23.22%, respectively; the remaining 10.62% consisted of other undesired movements. With micro-implant assistance, models B, C and D showed higher first molar mesialization efficiency than model A (values for each model: B = 52.92%, C = 55.04%, D = 53.80%), with the smallest increase in model B and the largest in model C. Consistent with greater applied traction, models B, C and D also displayed a higher proportion of undesired movements than model A. These results suggested that micro-implants could improve the first molar mesialization (especially buccal button), and reduce the second molar and first premolar distalization (especially angel buttons), but also had more undesired movement (especially angel buttons). Furthermore, with increasing traction force from 100 g to 200 g, the proportion of effective molar mesialization showed a gradual rise, indicating improved efficiency of targeted tooth movement. However, this was accompanied by a simultaneous increase in the proportion of unintended movements, suggesting that excessive force may compromise the quality of control and lead to less predictable outcomes.

### Three-dimensional displacement of the first molar

**Fig. 7:**
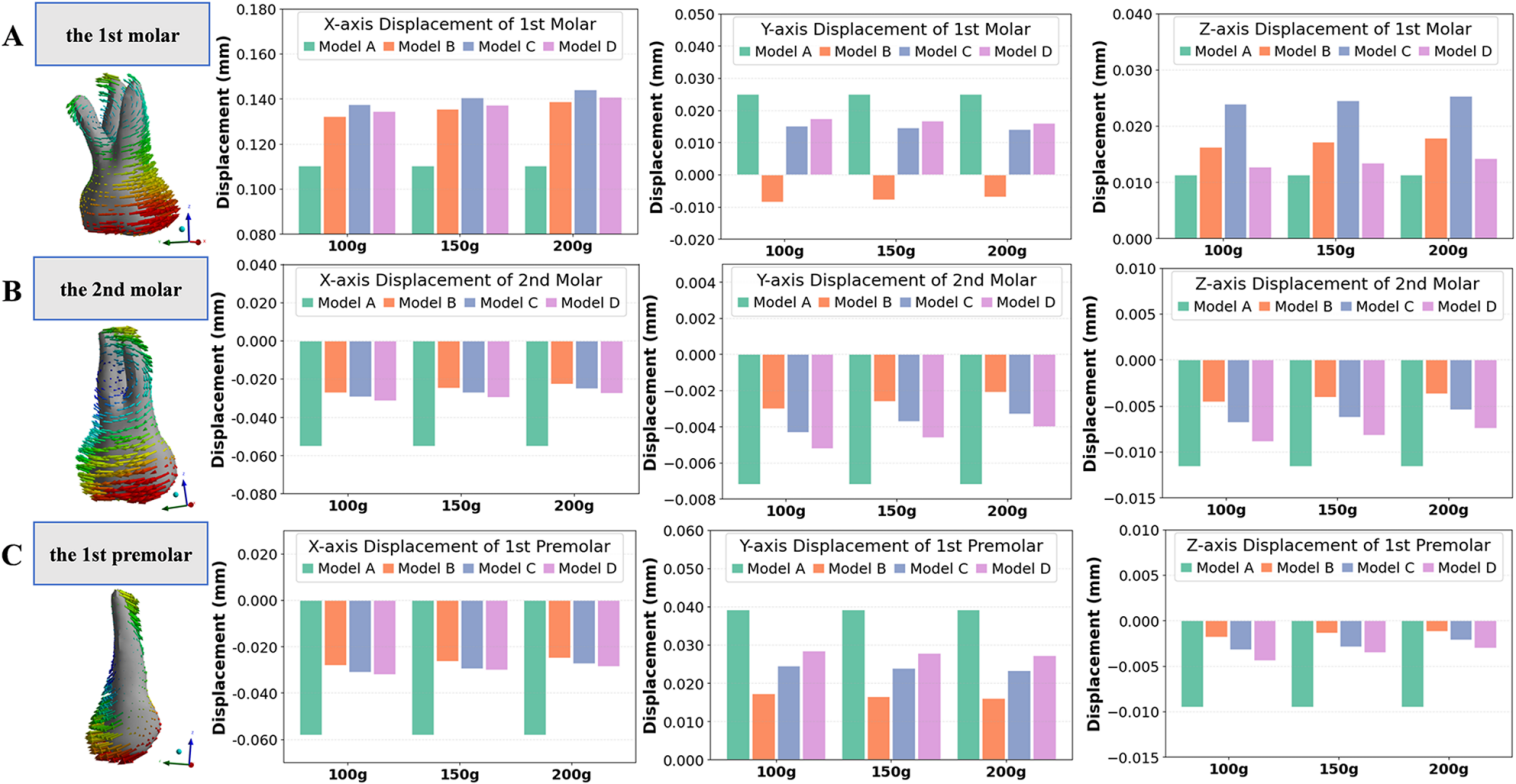
Three-dimensional displacement patterns of the first molar, second molar and first premolar according to the local coordinate system. **A** Three-dimensional displacement patterns of the first molar. **B** Three-dimensional displacement patterns of the second molar. **C** Three-dimensional displacement patterns of the first premolar. The histograms display displacement values along the x-, y-, and z-axis (mm) under varying traction forces. A local coordinate system is established for each tooth, where positive x-axis values correspond to the mesial direction, positive y-axis values indicate the palatal direction, positive z-axis values indicate the gingival direction. The first molar showed predominant mesial displacement (x+), with minimal transverse (y±) and intrusion (z+). Micro-implant assisted models increased mesial movement, with buccal buttons (model C) showing the most, followed by power arms (model D) and angel buttons (model B). The second molar showed predominant distal displacement (x-) with a small buccal shift (y-) and extrusion (z-). Micro-implant-assisted models reduced distal displacement (x-) and buccal shift (y-), with angel buttons showing the greatest reduction, followed by buccal buttons and then power arms. The first premolar similarly showed distal displacement (x-), with slight lingual shift (y+) and extrusion (z-). The effectiveness in reducing them was greatest in model B, followed by model C and D

The displacement patterns of the first molar varied considerably among the four models, as illustrated in Fig. 7A; Table 3. All models exhibited that the first molar primarily moved mesially, with lingual or buccal movement and intrusion. The magnitude of displacement showed a positive correlation with the intensity of the applied force in models B, C and D (Supplementary file 2). Due to the assistance of the micro-implant, the mesial movement of the first molar in models B, C and D was increased compared to model A. The increment in model B was the smallest, whereas model C exhibited the largest. The use of auxiliary attachments also reduced the mesial tipping tendency of the first molar compared with model A (0.831°), with model D showing the least tipping (at 100 g, 0.266°) and model C (at 100 g, 0.714°) the most. Moreover, the first molar experienced lingual movement (0.025 mm) and intrusion (0.0113 mm) during mesial movement with CA alone. With the assistance of the micro-implant, the lingual movement decreased and the intrusion of the first molar increased. Among the three models with auxiliary devices, the lingual movement in model C was the smallest (at 100 g, 0.0152 mm), while B displayed slight buccal movement. The first molar exhibited the greatest intrusion (at 100 g, 0.0239 mm) in model C, and the least in model D (at 100 g, 0.0127 mm).

**Table 3:** Tipping angle of the molar and premolar in the X-Z plane (+, mesial; -, distal)

		The first molar	The second molar	The first premolar
Model A		0.831°	-0.229°	-0.243°
Model B	100 g	0.512°	-0.088°	-0.092°
	150 g	0.536°	-0.094°	-0.103°
	200 g	0.557°	-0.112°	-0.109°
Model C	100 g	0.714°	-0.101°	-0.114°
	150 g	0.743°	-0.117°	-0.127°
	200 g	0.784°	-0.133°	-0.150°
Model D	100 g	0.266°	-0.120°	-0.139°
	150 g	0.273°	-0.125°	-0.143°
	200 g	0.289°	-0.132°	-0.151°

### Three-dimensional displacement of the second molar and first premolar

The second molar mainly exhibited distal displacement and tipping, with slight buccal movement and extrusion during the mesial movement of the first molar (Fig. 7B; Table 3). In model A, where no additional anchorage was provided, the second molar showed the most pronounced distal tipping (-0.229°), as well as the largest distal displacement, buccal movement and extrusion (-0.0551 mm, -0.0072 mm, and − 0.0116 mm, respectively). With the assistance of the micro-implant (100 g), the second molar exhibited reduced distal inclination (model B: -0.088°; model C: -0.101°; model D: -0.120°), buccal displacement (model B: -0.0030 mm; model C: -0.0043 mm; model D: -0.0052 mm), and extrusion (model B: -0.0046 mm; model C: -0.0068 mm; model D: -0.0089 mm). These reductions were most pronounced in model B and least evident in model D. These results suggested that all three auxiliary supported models effectively reduced undesired second molar movement, with model B providing the greatest stability, followed by model C and model D.

The first premolar also mainly showed distal displacement and tipping, accompanied by slight lingual movement and extrusion (Fig. 7C; Table 3). In model A, the premolar experienced the greatest distal movement (-0.0581 mm) and distal tipping (-0.243°), along with the largest lingual movement (0.0392 mm) and extrusion (-0.0095 mm). With the assistance of the micro-implant, distal tipping, lingual displacement, and extrusion of the first premolar were reduced. Under traction forces of 100 g, 150 g, and 200 g, the distal tipping angles were − 0.092°, − 0.103°, and − 0.109° in model B (angel button), − 0.114°, − 0.127°, and − 0.150° in model C (buccal button), and − 0.139°, − 0.143°, and − 0.151° in model D (power arm). These changes were most significant in model B and least pronounced in model D. The results indicated that all three models supported by auxiliaries effectively minimized unwanted premolar movement, with stability greatest in model B, intermediate in model C, and lowest in model D.

### Three-dimensional displacement of the anterior teeth and midline deviation

**Fig. 8:**
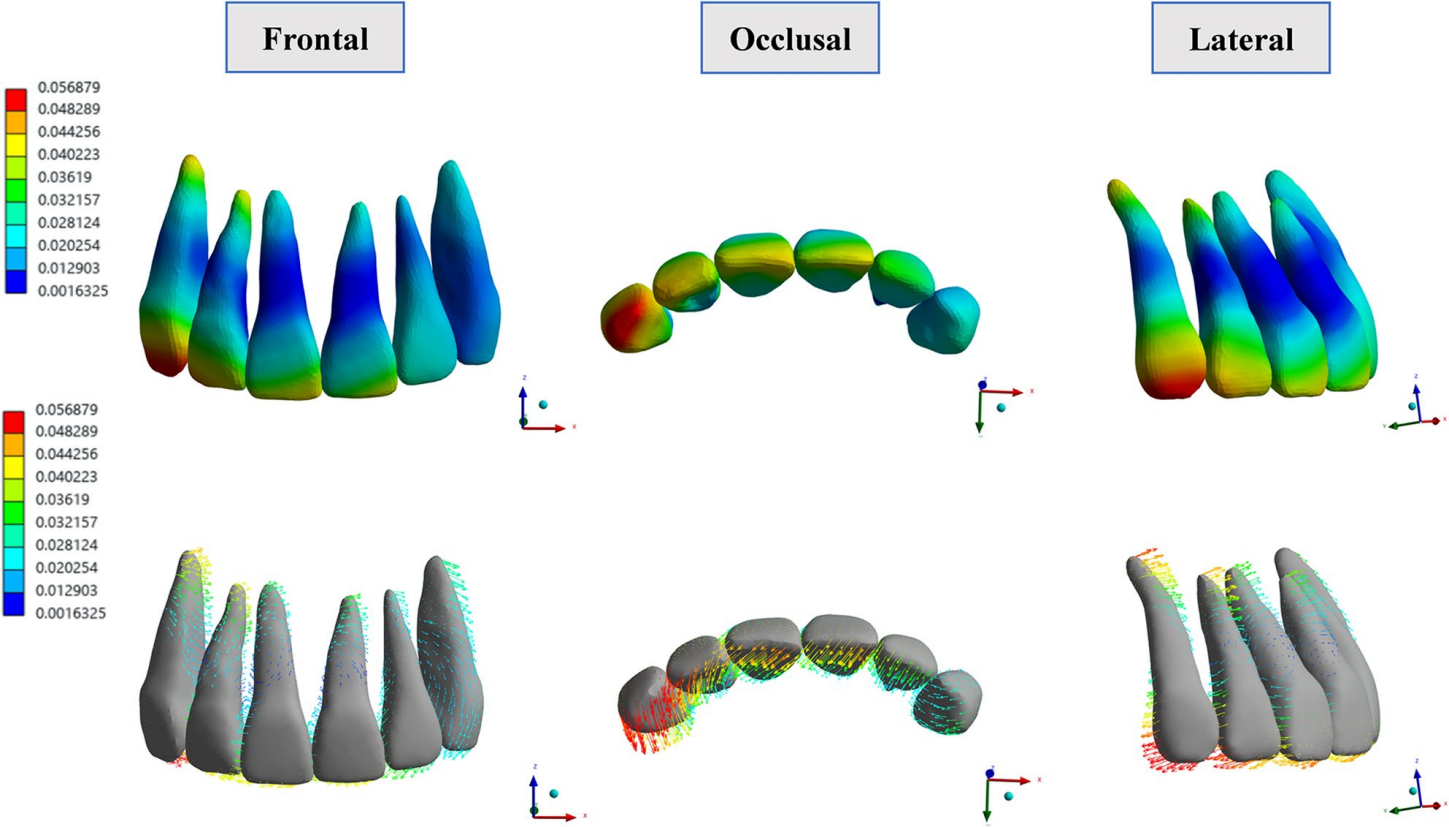
Displacement patterns of maxillary anterior anchorage teeth. Color maps and vector diagrams illustrate the displacement patterns of the anterior anchorage teeth. The anterior teeth showed a tendency to shift toward the edentulous side, accompanied by palatal tipping and slight extrusion

**Fig. 9:**
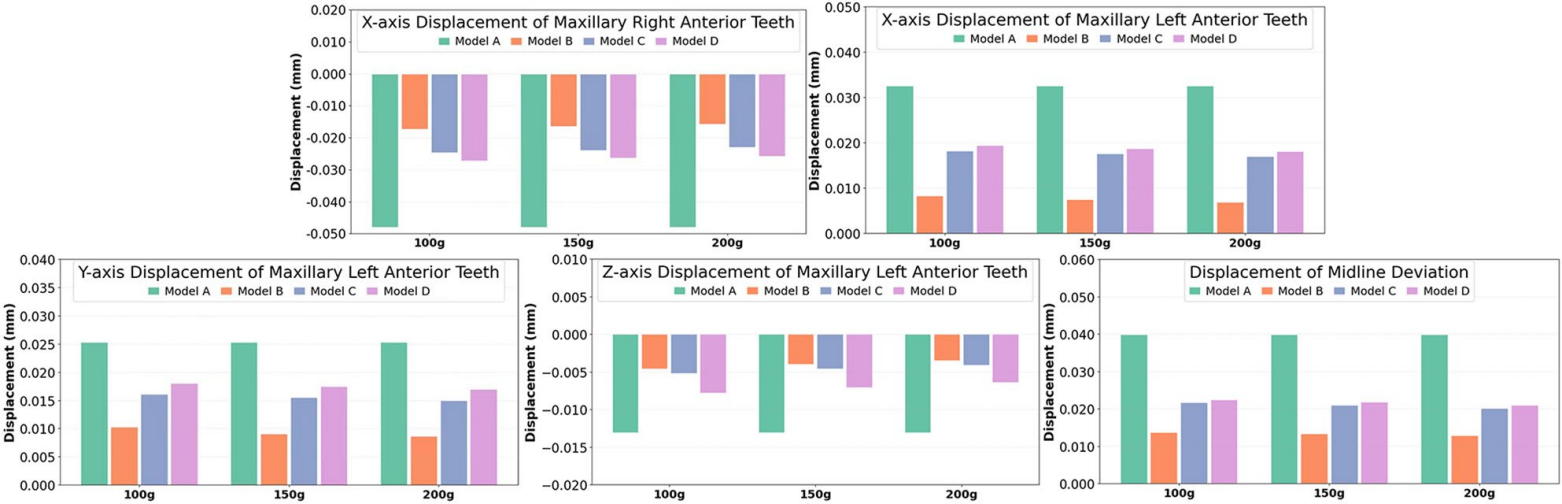
Three-dimensional displacement values for anterior anchorage teeth under different traction forces. Displacement values shown in the histograms are referenced to the local coordinate system (X axis: mesial; Y axis: palatal; Z axis: gingival). The midline deviation is referenced to the global coordinate system, with positive values indicating shifts to the patient’s right and negative to the left (relative to the anterior teeth midline). Compared with model A, models B-D reduced distal displacement, palatal shift, and extrusion; model B (angel button) produced the smallest anterior displacement, model C was intermediate, and model D was slightly greater than C. The arch midline deviated toward the edentulous side in all groups, with the least deviation in model B and slightly more in model D than in model C

The anterior teeth exhibited a tendency toward distopalatal tipping and extrusion (Figs. 8 and 9), resulting in anterior teeth retraction and midline deviation to the side of the edentulous space in all models. Compared to model A, all undesired anterior tooth movement (distal movement, palatal shift and extrusion) was reduced in models B, C, and D, with model B showing the least amount of anterior teeth displacement (at 200 g: distal displacement = -0.0157 mm; palatal = 0.0087 mm; extrusion = -0.0035 mm). Model D exhibited slightly more undesired anterior tooth movement than model C.

Moreover, the midline deviated toward edentulous space in all models (Supplementary file 3). In model A, the anterior segment showed the most pronounced distal displacement, with an average midline deviation of  0.0399 mm toward the traction side. In model B, the deviation of midline was the least, measuring only 0.0137 mm, indicating excellent anchorage preservation and minimal reciprocal movement. Model D had slightly more midline deviation than model C.

### Hydrostatic stress distribution in PDL

**Fig. 10:**
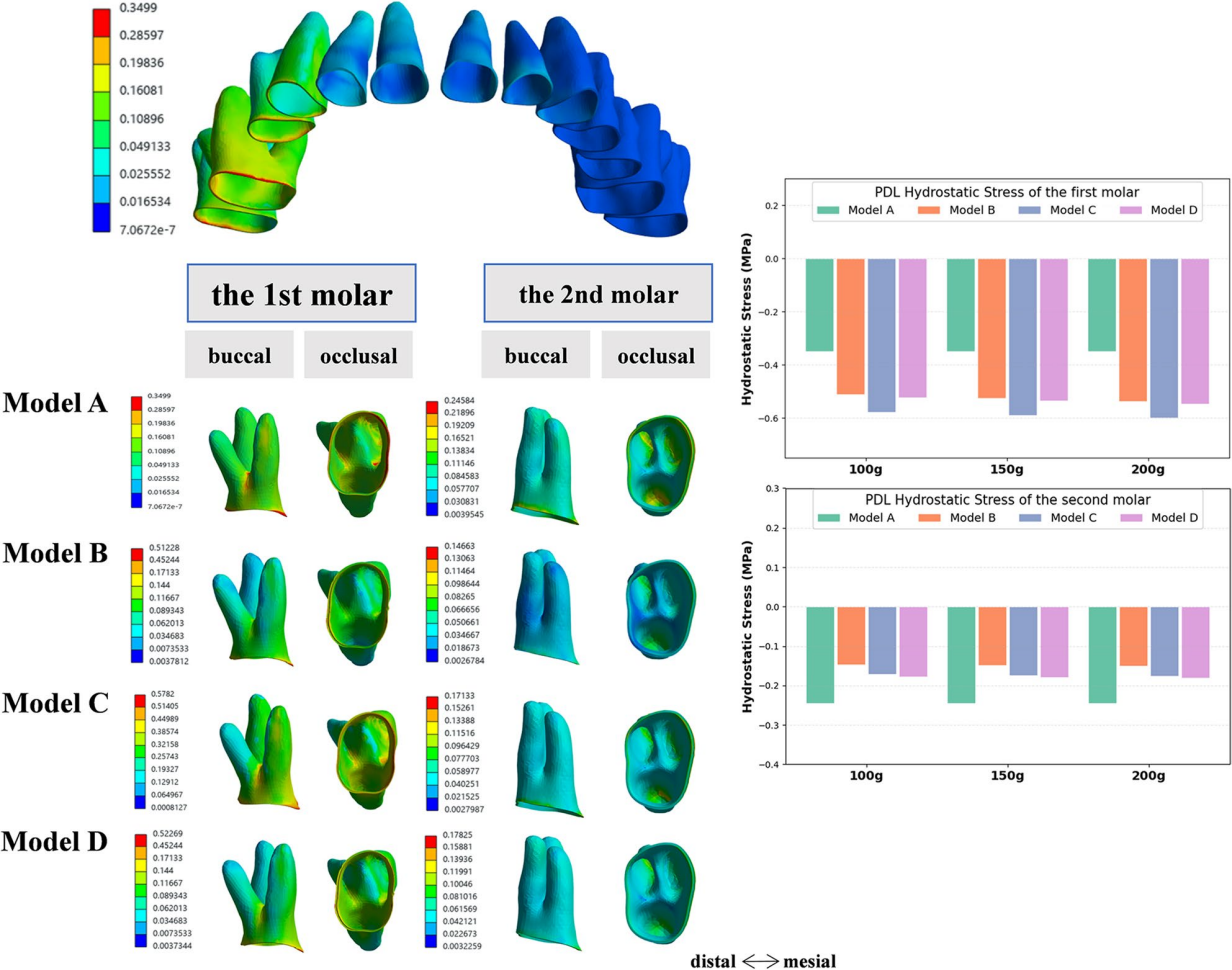
Hydrostatic stress distribution in the PDL. Color maps (buccal and occlusal views) show PDL hydrostatic pressure (MPa) for the whole dentition and molar region; histograms depict peak first-molar and second-molar values at 100, 150, and 200 g. In model A (CA alone), peaks were localized to the mesial cervical and furcation regions; distributions were most uniform in model D. With micro-implant assistance, the PDL hydrostatic stress in the second molar was reduced

The PDL hydrostatic pressure was evaluated using the dentition as the unit of analysis to better reflect the biomechanical response under different loading conditions (Fig. 10). For the first molar in model A, the highest hydrostatic pressure within the PDL was mainly localized in the mesial cervical region and the furcation area. Across the four models, the distribution of PDL hydrostatic pressure differed. Models A and C exhibited the most heterogeneous patterns, whereas model D showed the most uniform distribution. Simultaneously, the micro-implant raised the PDL hydrostatic pressure. Compared with model A, the maximum PDL pressure increased in models C, B, and D. The elevated peaks in model C were attributable to direct traction on the first molar, which concentrated a less even stress field.

In the second molar, the peak PDL hydrostatic pressure localized predominantly to the distal cervical region and was lower than that of the first molar. Compared to model A, models B, C, and D exhibited significantly reduced PDL hydrostatic stress. Notably, under increasing force (from 100 g to 200 g), the stress distribution remained stable, indicating better loading management and a reduced risk of tissue overload.

## Discussion

CAT has become a popular choice in orthodontic treatment. However, achieving effective molar mesialization with CA remains challenging due to limitations in force delivery and anchorage control. These challenges often result in unwanted tooth movements such as tipping, lingual inclination, and mesial tipping [31]. Additionally, the lack of rigid anchorage in CA may lead to reciprocal movements of adjacent teeth, reducing the overall efficiency of molar mesialization. These limitations highlight the need for adjunctive strategies, such as skeletal anchorage or force modulating auxiliaries, to improve the predictability and effectiveness of molar mesialization with CA. Recent studies on molar distalization have highlighted similar challenges with anchorage control. For instance, Oğuz et al. [32] examined the biomechanical effects of different anchorage locations for distalization, showing that force height and anchorage site significantly influence tooth movement patterns and stress distribution. Similarly, Guo et al. [33] compared various anchorage designs in molar distalization using CA, finding that anchorage reinforcement significantly improved tooth movement efficiency and reduced unwanted tipping and extrusion. Besides, a study reviewed that CA with a modified lever arm reduced undesigned mesial tipping and rotation, optimized molar mesialization biomechanics [17]. These studies underscored that both molar mesialization and distalization share anchorage control challenges. However, current research on molar mesialization using CA that combined auxiliary devices with micro-implants remains limited. Unlike previous finite element studies that focused on single anchorage control or individual auxiliary devices, this work directly compared the biomechanical effects of multiple auxiliaries, including aligner-based angel buttons, buccal buttons, and power arms combined with temporary anchorage devices. To the best of our knowledge, this is the first comparative approach to provide a comprehensive understanding of how different force delivery systems influence molar mesialization and anchorage control in CA.

**Fig. 11:**
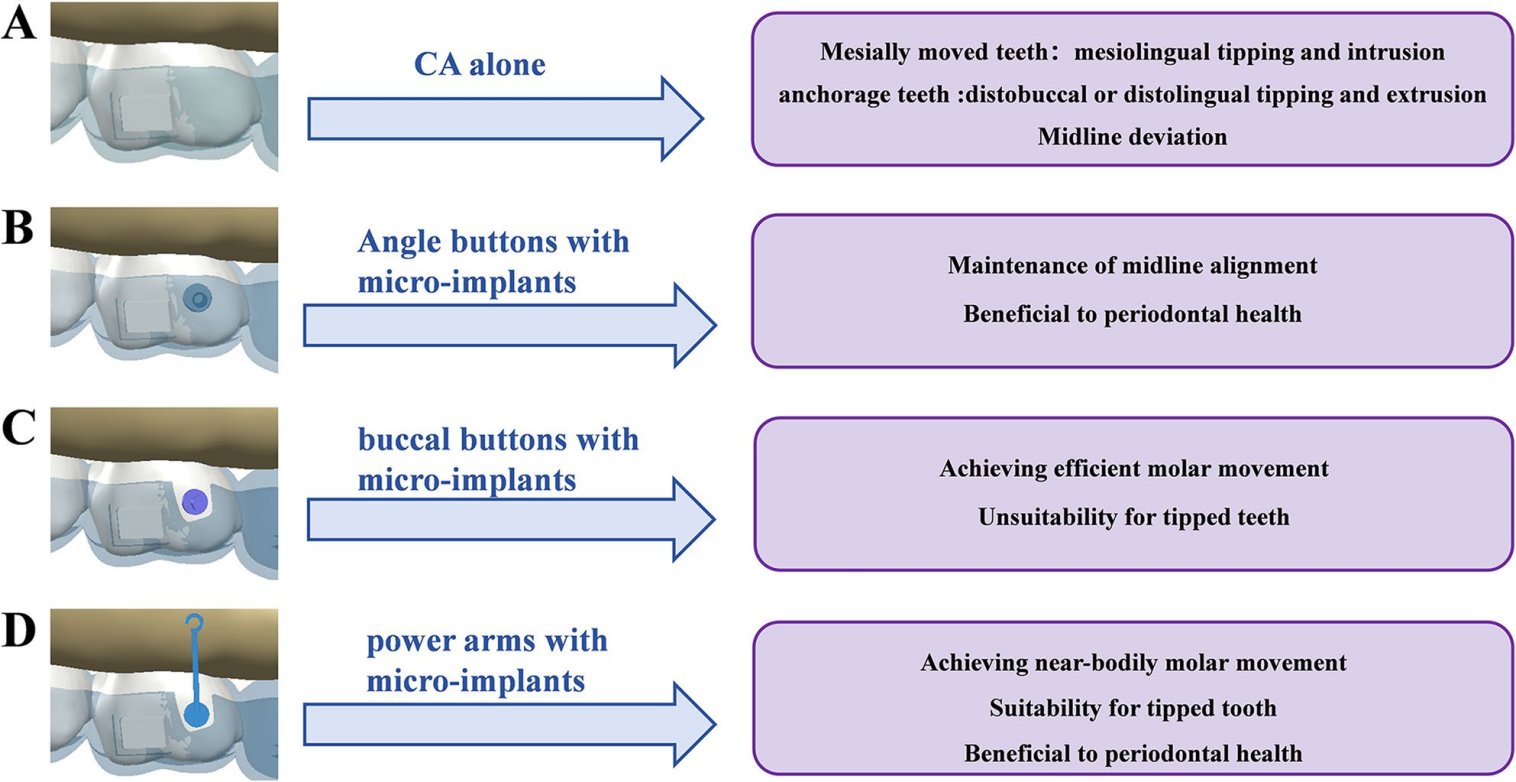
Summary of clinical application insights for four models. **A** CA alone: resulting in mesiolingual tipping and intrusion of mesially moved teeth, and anchorage loss with midline deviation. **B** Angle buttons with micro-implants: facilitating maintenance of midline alignment and periodontal health. **C** Buccal buttons with micro-implants: achieving efficient molar movement but unsuitable for tipped teeth. **D** Power arms with micro-implants: achieving near-bodily molar movement, suitable for tipped teeth, and beneficial to periodontal health

Our study revealed a key finding in CAT: when using CA to move the first molar mesially, the main tooth movement was the mesialization of the first molar, followed by the distalization of the second molar and the first premolar. At the same time, other movements such as the retraction of the anterior teeth and the tipping movement of the tooth also occurred (Fig. 11A). Micro-implant assistance improved the efficiency of first molar mesialization and increased its intrusion, while reducing its mesial inclination, minimizing the distal movement and lingual inclination of the anchoring teeth. These findings suggested that the use of micro-implants enhanced the efficiency of molar mesialization with CA and helped stabilize the anchorage teeth. In addition, treatment designs that counteracted mesial tipping and lingual displacement of the molars were necessary.

The tooth movement will be influenced by the direction of the traction force and the position of the resistance center [34, 35]. Gandhi et al. [35] identified the center of resistance of maxillary first molars is located apical and distal to the root trifurcation. As the center of rotation gets closer to the center of resistance, the molar demonstrates more parallel movement and less unwanted tipping and rotation [36]. In our study, all three auxiliary attachments including the aligner-based angel button, the buccal button and the power arm significantly influenced both the efficiency of first molar mesialization and the pattern of tooth movement. The aligner-based angel button distributed traction through CA, improving anchorage control and midline stability, but with less bodily movement than the power arm. The buccal button bonded to the first molar not only significantly increased mesialization efficiency, but also had the greatest effect on its intrusion and mesial inclination, making it unsuitable for already tipped molars (Fig. 11C). The power arm increased the height of the traction force and directed the force more closely toward the tooth’s center of resistance, helping reduce mesial tipping while still achieving mesialization, consistent with the findings of Hong et al. [37]. Consequently, the power arm is indicated for correcting pre-existing tooth tipping or for cases requiring near-bodily movement (Fig. 11D).

CAT for unilateral molar movement often requires attention to midline deviation. The alignment between the maxillary dental midline and facial midline plays a pivotal role in facial esthetics [38]. Wang et al. [39] demonstrated that a midline discrepancy within 2 mm was not visually identifiable by either dentists or untrained observers. In cases of unilateral tooth loss requiring molar mesialization, the reactive forces generated during molar mesialization often lead to a shift of the anterior teeth toward the edentulous side, resulting in midline deviation. Our simulations quantified this effect, revealing midline shifts of up to 0.0399 mm during the 0.25 mm first step in CA-only therapy. As treatment progresses, midline deviation might become more noticeable. When micro-implants were employed to deliver external forces, these undesired reactive effects could be effectively counteracted. Specifically, when using the aligner-based angel button, the traction force from the micro-implant was distributed through the entire CA system, thereby reducing the midline deviation to a magnitude of only 0.0137 mm. This design allowed for better force dissipation and anchorage control, thereby minimizing its influence on the dental midline, suitable for patients with high aesthetic demands for midline alignment or those with existing mild midline discrepancy (Fig. 11B). However, the force distribution through the CA resulted in less bodily movement and more crown tipping of the first molar compared to the power arm design.

The magnitude and location of PDL hydrostatic pressure are related to the type of tooth movement as well as the direction and magnitude of force [40]. In this study, the PDL hydrostatic stress of the first molar was mainly concentrated at the mesial cervical and furcation regions, demonstrating that tooth movement in all models was mainly tipping movement, which was consistent with the findings of Jain’s research [41]. Our research also showed that the use of micro-implants not only increased the PDL hydrostatic stress on the first molar, but also affected the uniformity of the stress distribution in the PDL. Similar results were also observed in studies on the molar distalization [12]. Furthermore, if the traction force applied by the micro-implant directly acted on the buccal button bonded to the buccal regions of the first molar, it significantly increased the tipping movement, as well as the stress levels at the mesial cervical and furcation regions. However, using the power arm, since it was close to the resistance center of the first molar, the degree of tooth inclination decreased, and the unevenness of the stress distribution in the PDL was also reduced. The elevated PDL hydrostatic stress values observed in our simulations exceeded the clinical threshold of pathological risks. Excessive stress in the PDL can lead to risks such as root resorption, tooth loosening and PDL damage [30, 42]. However, it should be noted that the simulated force represented the maximum load generated by CA, and the actual force exerted by CA decayed rapidly over time. Therefore, the risks might have been overestimated. During molar mesialization, using power arms bonded to the tooth or angel buttons connected to CA is more beneficial for dental health, particularly for patients with periodontal concerns or periodontitis.

This study has several limitations that should be considered when interpreting the results. Firstly, the model was generated from the CBCT of a single subject, which may not adequately capture individual anatomical variations such as differences in root morphology, bone density, and PDL characteristics. Additionally, the simulation environment differed from actual clinical conditions. All materials, including the PDL, were modeled as homogeneous, isotropic, and linearly elastic. Studies [43, 44] have shown that different PDL material properties mainly affect stress magnitude rather than actual tooth movement patterns. Also, simplifying the material properties of the alveolar bone to cancellous bone may lead to discrepancies in the absolute values of displacement and stress. Future work should incorporate more detailed cortical bone modeling to further optimize the quantitative data. Furthermore, the assumption of a rigidly fixed maxilla was a simplification that may overestimate clinical anchorage stability, as it did not simulate the subtle flexibility of the craniofacial skeleton or long-term alveolar bone remodeling. Besides, the use of fully tied constraints at the PDL-bone, bone-implant, and attachment-tooth contacts idealized the dentoalveolar system. This idealization increased system stiffness, likely underestimating anchorage-tooth displacement, extrusion, and tipping. Additionally, static loading conditions neglect the time-dependent force decay of CA and biological responses. Incorporating viscoelastic material properties for the PDL and long-term, dynamic loading cycles would provide more valuable insights into the full clinical trajectory of tooth movement. Clinical studies are necessary to validate and refine these findings before they can be widely applied in practice.

## Conclusion

This study aimed to analyze the biomechanical efficiency of molar mesialization and other tooth movements combined with CA. The findings showed that when CA alone was used to move the first molar mesially, the molar exhibited mesiolingual tipping and intrusion, while the anchorage teeth underwent distobuccal or distolingual tipping and extrusion. The midline deviated toward the side of the missing tooth as a result of reciprocal forces. Micro-implants could increase the effective contribution of molar mesialization, reduce mesiolingual tipping of the first molar, stabilize the anchorage teeth, minimize midline deviation, and optimize the distribution of PDL hydrostatic stress in the first molar. Power arms tended to exhibit more bodily movement and more uniform PDL hydrostatic stress distribution; aligner-based angel buttons generally enhanced anchorage teeth stability and reduced midline deviation but showed more crown tipping than power arms; buccal buttons increased mesial displacement but were accompanied by greater mesiolingual tipping and a more heterogeneous PDL stress pattern.

Importantly, these findings were derived from FEA representing short-term mechanical responses under idealized assumptions. Accordingly, the results offered theoretical insights for clinical planning and require confirmation in future experimental and clinical studies.

## Supplementary Information

Supplementary Material 1.

Supplementary Material 2.

Supplementary Material 3.

## Data Availability

The data supporting this study are available from the corresponding author upon reasonable request.

## Abbreviations

CA: Clear aligner
CAT: Clear aligner therapy
PDL: Periodontal ligament
FEA: Finite element analysis
CBCT: Cone beam computed tomography
