# Supplementary material for: Comparative effects of temporary anchorage devices combined with various auxiliary attachments on maxillary molar mesialization with clear aligners: a finite element analysis
Source: BMC Oral Health. 2026 Apr 1;26:840. doi: 10.1186/s12903-026-08187-9 (PMC13169540; doi:10.1186/s12903-026-08187-9)
Supplement: Supplementary file 1 — Supplementary Material 1. [file 12903_2026_8187_MOESM1_ESM.docx]

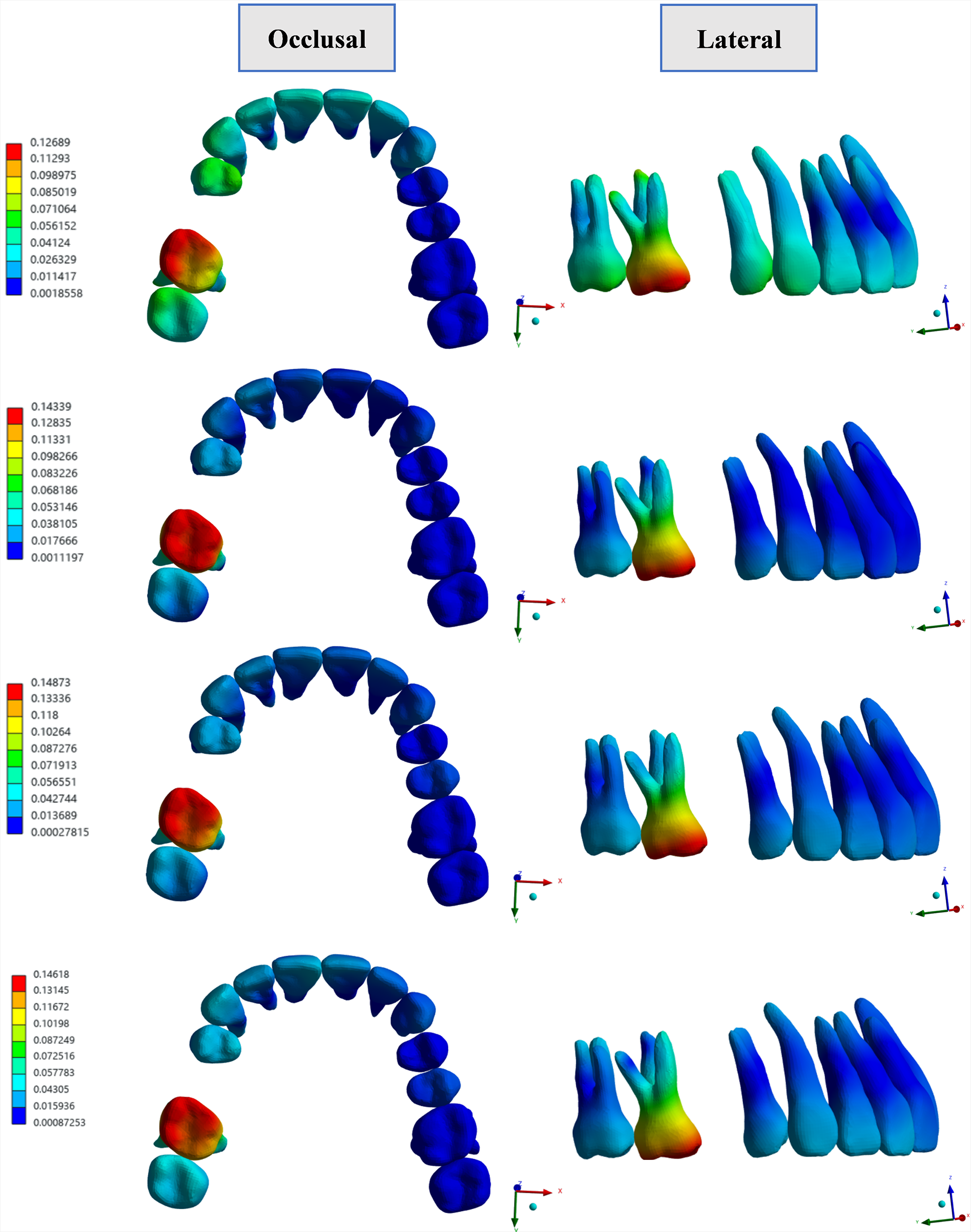


Supplementary file 1:Displacement pattern of the maxillary dentition in four models. In the color maps, red indicates regions of maximum displacement, while blue denotes areas of minimum displacement.
