# Supplementary material for: Comparative effects of temporary anchorage devices combined with various auxiliary attachments on maxillary molar mesialization with clear aligners: a finite element analysis
Source: BMC Oral Health. 2026 Apr 1;26:840. doi: 10.1186/s12903-026-08187-9 (PMC13169540; doi:10.1186/s12903-026-08187-9)
Supplement: Supplementary file 2 — Supplementary Material 2. [file 12903_2026_8187_MOESM2_ESM.docx]

**Supplementaryfile2.**Three-dimensional displacement for the posterior teeth(mm).

| Maxillary | Forcemagnitudes | Directions | X-axis | | | | Y-axis | | | | Z-axis | | | |
| --- | --- | --- | --- | --- | --- | --- | --- | --- | --- | --- | --- | --- | --- | --- |
|  |  | Models | A | B | C | D | A | B | C | D | A | B | C | D |
| The first molar | 100g | Crown | 0.1103±5.26% | 0.1323±4.71% | 0.1376±6.36% | 0.1345±3.02% | 0.0250±4.85% | -0.0084±8.68% | 0.0152±8.71% | 0.0175±8.25% | 0.0113±7.46% | 0.0163±3.90% | 0.0239±7.68% | 0.0127±5.59% |
|  |  | Root | -0.0314±4.98% | 0.0325±5.93% | 0.0124±12.21% | 0.0842±6.94% | -0.0030±9.12% | -0.0021±9.12% | -0.0013±9.98% | -0.0009±9.92% | 0.0112±7.35% | 0.0159±4.32% | 0.0240±7.47% | 0.0125±5.43% |
|  | 150g | Crown | 0.1103±5.26% | 0.1356±4.70% | 0.1405±6.93% | 0.1374±3.78% | 0.0250±4.85% | -0.0077±9.04% | 0.0147±8.45% | 0.0168±7.90% | 0.0113±7.46% | 0.0172±3.44% | 0.0245±7.66% | 0.0134±5.90% |
|  |  | Root | -0.0314±4.98% | 0.0326±5.82% | 0.0125±12.35% | 0.0839±7.13% | -0.0030±9.12% | -0.0019±9.44% | -0.0014±9.76% | -0.0010±9.88% | 0.0112±7.35% | 0.0172±3.44% | 0.0242±7.45% | 0.0132±5.87% |
|  | 200g | Crown | 0.1103±5.26% | 0.1389±5.04% | 0.1442±6.41% | 0.1407±3.27% | 0.0250±4.85% | -0.0069±9.36% | 0.0141±8.66% | 0.0160±7.93% | 0.0113±7.46% | 0.0179±3.61% | 0.0253±7.83% | 0.0142±5.41% |
|  |  | Root | -0.0314±4.98% | 0.0323±5.33% | 0.0124±12.29% | 0.0840±7.13% | -0.0030±9.12% | -0.0020±9.36% | -0.0016±9.68% | -0.0011±9.75% | 0.0112±7.35% | 0.0179±3.61% | 0.0250±7.78% | 0.0141±5.40% |
| The second molar | 100g | Crown | -0.0551±4.83% | -0.0272±5.12% | -0.0293±5.23% | -0.0314±3.26% | -0.0072±6.22% | -0.0030±7.25% | -0.0043±7.14% | -0.0052±5.43% | -0.0116±6.24% | -0.0046±4.13% | -0.0068±6.25% | -0.0089±5.12% |
|  |  | Root | -0.0116±5.77% | 0.0051±4.58% | 0.0069±5.46% | 0.0174±3.45% | 0.0035±13.48% | 0.0017±15.10% | -0.0128±5.22% | -0.0178±4.91% | -0.0115±5.94% | -0.0043±4.02% | -0.0067±6.22% | -0.0087±5.18% |
|  | 150g | Crown | -0.0551±4.83% | -0.0250±5.22% | -0.0273±5.66% | -0.0298±3.23% | -0.0072±6.22% | -0.0026±7.23% | -0.0037±6.89% | -0.0046±5.48% | -0.0116±6.24% | -0.0041±4.93% | -0.0062±5.83% | -0.0082±5.93% |
|  |  | Root | -0.0116±5.77% | 0.0093±4.58% | 0.0087±5.59% | 0.0088±3.54% | 0.0035±13.48% | 0.0024±13.93% | -0.0144±6.16% | -0.0188±4.99% | -0.0115±6.44% | -0.0039±4.88% | -0.0064±5.77% | -0.0079±5.79% |
|  | 200g | Crown | -0.0551±4.83% | -0.0228±5.64% | -0.0253±5.54% | -0.0277±3.15% | -0.0072±6.22% | -0.0021±7.66% | -0.0033±6.73% | -0.0040±5.63% | -0.0116±6.24% | -0.0037±5.03% | -0.0054±6.13% | -0.0074±5.26% |
|  |  | Root | -0.0116±5.77% | 0.0110±4.58% | 0.0098±5.61% | 0.0099±3.46% | 0.0035±13.48% | 0.0014±18.05% | -0.0151±6.24% | -0.0192±5.90% | -0.0115±6.32% | -0.0035±4.95% | -0.0052±5.09% | -0.0071±5.14% |
| The first premolar | 100g | Crown | -0.0581±6.85% | -0.0281±4.58% | -0.0311±5.95% | -0.0320±4.64% | 0.0392±5.53% | 0.0173±9.01% | 0.0245±9.13% | 0.0285±7.53% | -0.0095±6.16% | -0.0018±5.14% | -0.0032±7.63% | -0.0044±6.16% |
|  |  | Root | 0.0192±5.92% | -0.0064±5.89% | -0.0064±6.58% | -0.0016±9.99% | 0.0147±7.11% | 0.0086±10.28% | 0.0096±8.37% | 0.0130±6.85% | -0.0099±6.23% | -0.0015±4.62% | -0.0030±7.88% | -0.0041±6.23% |
|  | 150g | Crown | -0.0581±6.85% | -0.0264±4.82% | -0.0295±5.39% | -0.0301±4.82% | 0.0392±5.53% | 0.0165±8.94% | 0.0239±8.82% | 0.0279±7.56% | -0.0095±6.16% | -0.0014±5.90% | -0.0029±7.55% | -0.0035±6.24% |
|  |  | Root | 0.0192±5.92% | -0.0065±6.21% | -0.0064±6.43% | -0.0017±9.99% | 0.0147±7.11% | 0.0076±12.58% | 0.0091±8.54% | 0.0126±6.75% | -0.0099±6.23% | -0.0011±5.94% | -0.0025±7.54% | -0.0036±6.28% |
|  | 200g | Crown | -0.0581±6.85% | -0.0248±4.93% | -0.0274±5.83% | -0.0285±4.96% | 0.0392±5.53% | 0.0160±8.02% | 0.0233±8.78% | 0.0272±7.73% | -0.0095±6.16% | -0.0012±5.88% | -0.0021±7.23% | -0.0030±6.25% |
|  |  | Root | 0.0192±5.92% | -0.0063±6.34% | -0.0065±6.52% | -0.0016±9.99% | 0.0147±7.11% | 0.0072±8.53% | 0.0087±8.56% | 0.0118±6.47% | -0.0099±6.23% | -0.0012±5.88% | -0.0023±7.11% | -0.0031±6.17% |

Thecoordinatesystemwascenteredoneachtooth(localcoordinatesystem).Apositivevalueonthex-axisrepresentsthemesialsurfaceoftheteeth,apositivevalueonthey-axisrepresentsthelingualsurfaceoftheteeth,andapositivedirectiononthez-axisrepresentstowardstheapexofthemaxillaryteeth.
