# Supplementary material for: Comparative effects of temporary anchorage devices combined with various auxiliary attachments on maxillary molar mesialization with clear aligners: a finite element analysis
Source: BMC Oral Health. 2026 Apr 1;26:840. doi: 10.1186/s12903-026-08187-9 (PMC13169540; doi:10.1186/s12903-026-08187-9)
Supplement: Supplementary file 3 — Supplementary Material 3. [file 12903_2026_8187_MOESM3_ESM.docx]

**Supplementaryfile3.** Comparison of tooth movement among the four Models

|  | | Model A | | | Model B | | | Model C | | | Model D | | |
| --- | --- | --- | --- | --- | --- | --- | --- | --- | --- | --- | --- | --- | --- |
|  |  | 100g | 150g | 200g | 100g | 150g | 200g | 100g | 150g | 200g | 100g | 150g | 200g |
| The first molar | Mesialization efficiency（%） | 44.12% | 44.12% | 44.12% | 52.92% | 54.24% | 55.56% | 55.04% | 56.20% | 57.68% | 53.80% | 54.96% | 56.28% |
|  | Lingual shift（mm） | 0.0250 | 0.0250 | 0.0250 | -0.0084 | -0.0077 | -0.0069 | 0.0152 | 0.0147 | 0.0141 | 0.0175 | 0.0168 | 0.0160 |
|  | Intrusion (mm) | 0.0113 | 0.0113 | 0.0113 | 0.0163 | 0.0172 | 0.0179 | 0.0239 | 0.0245 | 0.0253 | 0.0127 | 0.0134 | 0.0142 |
|  | Tipping angle (°) | 0.831° | 0.831° | 0.831° | 0.512° | 0.536° | 0.557° | 0.714° | 0.743° | 0.784° | 0.266° | 0.273° | 0.289° |
| The second molar | Distalization  Contribution（%） | 22.04% | 22.04% | 22.04% | 10.88% | 10.01% | 9.11% | 11.72% | 10.91% | 10.12% | 12.56% | 11.91% | 11.06% |
|  | Buccal shift（mm） | 0.0072 | 0.0072 | 0.0072 | 0.0030 | 0.0026 | 0.0021 | 0.0043 | 0.0037 | 0.0033 | 0.0052 | 0.0046 | 0.0040 |
|  | Extrusion（mm） | 0.0116 | 0.0116 | 0.0116 | 0.0046 | 0.0041 | 0.0037 | 0.0068 | 0.0062 | 0.0054 | 0.0089 | 0.0082 | 0.0074 |
|  | Tipping angle (°) | -0.229° | -0.229° | -0.229° | -0.088° | -0.094° | -0.112° | -0.101° | -0.117° | -0.133° | -0.120° | -0.125° | -0.132° |
| The first premolar | Distalization  Contribution（%） | 23.22% | 23.22% | 23.22% | 11.25% | 10.54% | 9.90% | 12.43% | 11.80% | 10.96% | 12.81% | 12.03% | 11.40% |
|  | Lingual shift（mm） | 0.0392 | 0.0392 | 0.0392 | 0.0173 | 0.0165 | 0.0160 | 0.0245 | 0.0239 | 0.0233 | 0.0285 | 0.0279 | 0.0272 |
|  | Extrusion（mm） | 0.0095 | 0.0095 | 0.0095 | 0.0018 | 0.0014 | 0.0012 | 0.0032 | 0.0029 | 0.0021 | 0.0044 | 0.0035 | 0.0030 |
|  | Tipping angle (°) | -0.243° | -0.243° | -0.243° | -0.092° | -0.103° | -0.109° | -0.114° | -0.127° | -0.150° | -0.139° | -0.143° | -0.151° |
| Midline deviation （mm） | | 0.0399 | 0.0399 | 0.0399 | 0.0137 | 0.0133 | 0.0129 | 0.0217 | 0.0210 | 0.0202 | 0.0225 | 0.0218 | 0.0210 |
| Anterior teeth | Palatal shift （mm） | 0.0253 | 0.0253 | 0.0253 | 0.0103 | 0.0091 | 0.0087 | 0.0161 | 0.0155 | 0.0150 | 0.0180 | 0.0175 | 0.0170 |
|  | Extrusion （mm） | 0.0131 | 0.0131 | 0.0131 | 0.0046 | 0.0040 | 0.0035 | 0.0052 | 0.0046 | 0.0041 | 0.0078 | 0.0071 | 0.0064 |
